# IL-27 as a novel biomarker for pruritus in nodular prurigo and bullous pemphigoid

**DOI:** 10.3389/fimmu.2024.1499868

**Published:** 2024-12-13

**Authors:** Yifei Wang, Xin Zhang, Yun Te Teng, Chen Shen

**Affiliations:** ^1^ Department of Dermatology, Union Hospital, Tongji Medical College, Huazhong University of Science and Technology, Wuhan, China; ^2^ Department of Dermatology, Zhongnan Hospital of Wuhan University, Wuhan University, Wuhan, China

**Keywords:** bullous pemphigoid (BP), prurigo nodularis (PN), pruritus mechanisms, il-27, type 2 inflammation

## Abstract

**Introduction:**

Bullous pemphigoid (BP) and prurigo nodularis (PN) are chronic pruritic skin diseases that severely impact patients’ quality of life. Despite the widespread attention these two diseases have garnered within the dermatological field, the specific pathogenesis, particularly the molecular mechanisms underlying the pruritus, remains largely unclear. Limited clinical sequencing studies focusing on BP and PN have hindered the identification of pathological mechanisms and the exploration of effective treatment strategies.

**Methods:**

To address this gap, we collected a total of 23 peripheral blood mononuclear cell samples from BP and PN patients, as well as healthy controls, and performed RNA sequencing analysis. By integrating bioinformatics and machine learning techniques, we aimed to uncover the shared immune regulatory networks and pruritus-related mechanisms between BP and PN.

**Results:**

Our study identified 161 differentially expressed genes shared between BP and PN, which were primarily enriched in immune activation and neural pathways, providing crucial molecular insights into the pruritus-related mechanisms of both diseases. Furthermore, using the machine learning algorithms of support vector machines and random forest, we pinpoint 7 crucial genes shared between the BP and PN databases. Among these, IL-27 emerged as a potential pivotal gene, as its mRNA expression levels strongly correlated with clinical parameters including pruritus scores, immunoglobulin E levels, and eosinophil counts. Validation experiments conducted on clinical samples from an additional 22 participants confirmed the upregulation of IL-27 expression in both BP and PN lesions.

**Discussion:**

This study is the first to unveil the shared inflammatory and immune pathways common to BP and PN, highlighting the critical role of IL-27 in the pathogenesis of these conditions. Our findings not only enhance the understanding of the intricate relationship between BP and PN, but also provide a foundation for the development of novel therapeutic strategies targeting these two dermatological conditions.

## 1 Introduction

Pruritus, commonly known as itching, is defined as an uncomfortable sensation that triggers the urge to scratch. When pruritus persists for more than six weeks, it is classified as chronic pruritus, which can significantly impact patient’s quality of life by disrupting sleep, social interactions, and work capacity (1, 2). Type 2 inflammatory skin diseases, such as atopic dermatitis (AD), prurigo nodularis (PN), and bullous pemphigoid (BP), are frequently associated with varying degrees of pruritus (3). Understanding the mechanism of itching is essential for developing effective treatments for skin diseases. However, current research has primarily focused on AD, the most prevalent Type 2 inflammatory skin disease (4–8). For instance, one study suggests that epithelial cells directly communicate to cutaneous sensory neurons via thymic stromal lymphopoietin in AD to promote itch (9). Additionally, protease-activated receptor-2 signaling has been shown to drive several levels of neuro-epidermal communication in AD (10), and itch-related genetics has been analyzed using RNA sequencing (RNA-seq) of AD lesional skin (11). Further exploration is needed into the mechanisms and treatment strategies for intractable itching conditions like PN and BP.

BP is the most common autoimmune subepidermal bullous disease, characterized by generalized pruritic urticaria-like plaques and tense subepidermal blisters (12, 13). Cytokines such as interleukin (IL)-5, IL-6, IL-10 are elevated in serum and blister fluids of BP patients (14). PN, recognized as one of the most severe pruritic skin diseases, presents with localized or generalized pruritus accompanied by intensely pruritic nodules (15). Despite some progress in managing pruritus symptoms, current treatments often fail to provide complete relief (16). The mechanisms of these diseases, especially their persistent pruritus, are unclear, limiting the development of effective treatments. Thus, investigating pruritus mechanisms is vital to enhance patient quality of life and clinical outcomes.

Existing studies suggest that PN and BP may share common pathologic mechanisms underlying the elicitation of pruritus. For instance, single-cell RNA sequencing of blister samples and surrounding erythematous lesions from BP patients has identified Th2 cells as the most critical immune cell subset, with the IL13-IL13 receptor A1 ligand-receptor pair playing a critical role in immune-stromal crosstalk in BP (17). Similarly, RNA-seq and differentially expressed gene (DEG) functional enrichment analysis on patient’s skin biopsies, revealed the enrichment of IL-4 and IL-13 signaling pathways, suggesting shared pathogenic mechanism between BP and PN (18). Also, Hiraiwa et al. reported that scratching and localized inflammation in PN lead to the exposure of new epitopes on the basement membrane, thereby generating the production of bullous pemphigoid antibodies, further supporting a potential correlation between PN and BP (19). However, most studies to date have focused on the exploring these two diseases individually, while overlooking potential common or correlating mechanisms that might exist in between.

In regards to the study of pruritus mechanisms in dermatoses, the role of serum biomarkers should not be overlooked. As an easily accessible tool, serum biomarkers can serve as direct and effective diagnostic and monitoring indicators in clinical practice (3). Additionally, multiple cytokines partially secreted by peripheral blood mononuclear cells (PBMCs), play an essential role in neuroimmune circuits and pruritus (8, 20, 21). Type 2 cytokines have been shown to activate mouse and human dorsal root ganglion neurons, highlighting the essential role in the development of chronic itch (22). Furthermore, plasma IL-31 and oncostatin M, both dysregulated Type 2 inflammatory biomarkers, have shown a strong correlation with pruritus, and biologics targeting IL-31 receptor A have achieved clinical success in both AD and PN (23–27). Therefore, the peripheral serum and PBMCs, acting as a crucial link between inflammation and pruritus sensation, may play a key role in elucidating the systemic pathological processes of pruritus (4).

Based on these considerations, we have shifted our research focus to PBMCs for the first time. By systematically comparing the gene expression patterns of PBMCs in PN and BP patients with those in healthy controls (HCs) through transcriptome sequencing, this study aims to explore the potential associations between these two diseases and their shared and unique molecular mechanisms. By identifying novel potential serum biomarkers and key molecular mechanisms, this study aims to provide new perspectives on the diagnosis, monitoring, and treatment of BP and PN.

## 2 Materials and methods

### 2.1 Participants information

This study included two independent cohorts, each consisting of patients with BP and PN, and HCs. All participants underwent thorough examination and were diagnosed by two experienced dermatologists and one skilled dermatopathologist. The diagnoses of BP and PN were based on the established guidelines, combining with clinical examination and laboratory investigations (28, 29). Exclusion criteria included: (1) pregnancy or breastfeeding; (2) presence of other known systemic inflammatory diseases, autoimmune disorders or infections; (3) treatment with any topical medications within 2 weeks, systemic medications within 4 weeks, and biologics within 12 weeks. Samples from the two cohorts were used for screening and validation of candidate biomarkers, respectively (**Table 1**). This study has been approved by the Ethics Committee of Wuhan Union Hospital, is consistent with the Helsinki Declaration II, and has obtained written informed consent from all participants.

**Table 1:** Characteristics of study participants.

Characteristic	Discover set			Validation set		
	BP n=6	PN n=9	HC n=8	BP n=7	PN n=10	HC n=5
Age (years), mean ± SD	64.67 ± 8.43	59.56 ± 7.73	52.00 ± 20.07	54.85 ± 10.93	52.90 ± 18.50	54.20 ± 14.34
Female, n (%)	2 (33.33)	6 (66.67)	4 (50.00)	3 (42.86)	3 (30.00)	2 (40)
Body mass index (kg/m^2^), mean ± SD	23.38 ± 4.35	23.02 ± 2.26	22.51 ± 1.25	23.72 ± 3.22	24.12 ± 1.91	22.62 ± 1.32
Disease duration, mean ± SD	1.03 ± 0.68	3.81 ± 2.89	–	1.30 ± 0.56	4.08 ± 2.92	–
P-NRS, mean ± SD	5.67 ± 2.16	4.67 ± 2.24	0	6.86 ± 1.35	6.10 ± 1.85	0
EOS (G/L), mean ± SD	0.24 ± 0.20	0.28 ± 0.18	0.17 ± 0.20	0.31 ± 0.16	0.36 ± 0.21	0.19 ± 0.08
IgE (IU/ml), mean ± SD	118.90 ± 121.27	310.95 ± 767.23	56.81 ± 55.76	177.10 ± 142.25	241.95 ± 170.65	67.97 ± 55.00

BP, bullous pemphigoid; PN, prurigo nodularis; HC, Health control; P-NRS, pruritus-numeric rating scale; EOS, eosinophil counts; IgE, immunoglobulin E.

### 2.2 PBMC isolation and RNA extraction

Blood from participants was collected in heparin tubes. PBMCs were isolated using Lymphocyte Separation Medium (Corning, Manassas, VA) via centrifugation. Cell counts were performed using the Cellometer Auto 2000 (Nexcelom, Lawrence, MA) and cells were cultured at 2 × 10^6^ cells/ml. RNA was extracted from PBMCs using TRIzol (Invitrogen, Carlsbad, CA). The quality of RNA was measured using NanoDrop ND-1000 (ThermoFisher Scientific, Waltham, MA). All raw data have been deposited in the GEO database under accession number GSE278382.

### 2.3 Differential expression analysis

Differential expression analysis of BP and PN samples with normal controls was performed using GEOquery and the limma package in R software. DEGs were identified based on the criteria of adjusted *P* < 0.05 and |log2FC| > 1. Volcano plots and heatmaps were generated to visualize DEGs in the BP and PN cohorts using the ‘heatmap’ and ‘ggplot2’ packages. Venn diagram software was used to identify the common DEGs between BP and PN samples.

### 2.4 Pathway enrichment analysis

Gene set variation analysis was performed to evaluate pathway enrichment in BP and PN datasets. All hallmark gene sets were downloaded from the Molecular Signature Database (MSigDB). An adjusted *P* value < 0.05 was considered statistically significant. The Benjamini and Hochberg method was used for multiple-testing adjustments.

### 2.5 Function enrichment analysis of DEGs

We utilized the Gene Ontology (GO) plot package and cluster Profiler in R for GO functional and Kyoto Encyclopedia of Genes and Genomes (KEGG) pathway analyses to gain deeper insights into the roles of hub genes in BP and PN. Annotation terms with a *P*-value < 0.05 were considered significantly enriched, and the final results were presented in a bubble diagram for clear visualization.

### 2.6 Protein-protein interaction network analysis

The protein-protein interaction (PPI) networks of DEGs were constructed using the STRING database. Interactions with a combined score greater than 0.4 were considered statistically significant. The PPI networks were visualized using Cytoscape software.

### 2.7 Selection and functional analysis of hub genes

Using the cytoHubba plugin of the Cytoscape software, hub genes were selected with the following selection criteria: K-core=2, degree cutoff=2, max depth=100, and node score cutoff=0.2. A co-expression network of these hub genes was constructed by GeneMANIA.

### 2.8 Selection of key genes through machine learning methods

Key genes linking BP and PN were identified using machine learning methods, including Random Forest (RF) and Support Vector Machine (SVM) algorithms. The former used the R package “randomForest”, and the latter utilized the R package “SVM”.

### 2.9 Receiver operating characteristic curve analysis

We used the receiver operating characteristic (ROC) function in the R package to perform ROC analysis. The area under the curve (AUC) of ROC was determined to validate key genes and assess their diagnostic value.

### 2.10 Gene set enrichment analysis

To investigate the different immune cell types and states in BP and PN, gene set enrichment analysis (GSEA) was performed by our differential gene expression data. MSigDB as the reference gene set was used as the reference gene set for gene enrichment and visualization.

### 2.11 Enzyme-linked immunosorbent assay and quantitative reverse transcription-polymerase chain reaction

Serum IL-27 levels were detected by commercial enzyme-linked immunosorbent assay (ELISA) kit. Total RNA was extracted from participants’ PBMCs by TRIzol^®^ reagent (Thermo Fisher Scientific, Inc.) based on the manufacturer’s instructions. After that reverse transcription was performed with the PrimeScript™ RT reagent Kit (Takara Bio, Inc.), and quantitative reverse transcription-polymerase chain reaction (qRT-PCR) was performed via real-time quantitative PCR with SYBR Green Real-Time PCR Master Mixes (Applied Biosystems). mRNA expression levels were quantified applying the 2−ΔΔCq method and normalized to ACTB. All primer sequences were presented in **Table 2**.

**Table 2:** The primer sequences of IL-27 and ACTB.

Gene	Forward primer (5’ to 3’)	Reverse primer (5’ to 3’)
IL-27	CTTGGCTGGCGGCTCA	CCAAAGTGTAGGTCCCTGGC
ACTB	GCCGCCAGCTCACCAT	GCTGACTGTGAACTCCCTCC

### 2.12 Immunohistochemistry

Tissue microarray slides were purchased from Xi’an Avili Biotechnology Co., Ltd. (Xi’an, China) (DC-Hea11012). PN and BP samples used for immunohistochemistry (IHC) were primarily collected from lesional skin on the limbs. Tissue microarray specimens were immunostained with IL-27. The extent of immunostaining was reviewed and scored by two independent pathologists who were blinded to the clinical details. The score was determined by multiplying the percentage of positive cells by the staining intensity.

## 3 Result

### 3.1 Clinical characteristics of the participants and identification of DEGs in BP and PN

**Figure 1:**
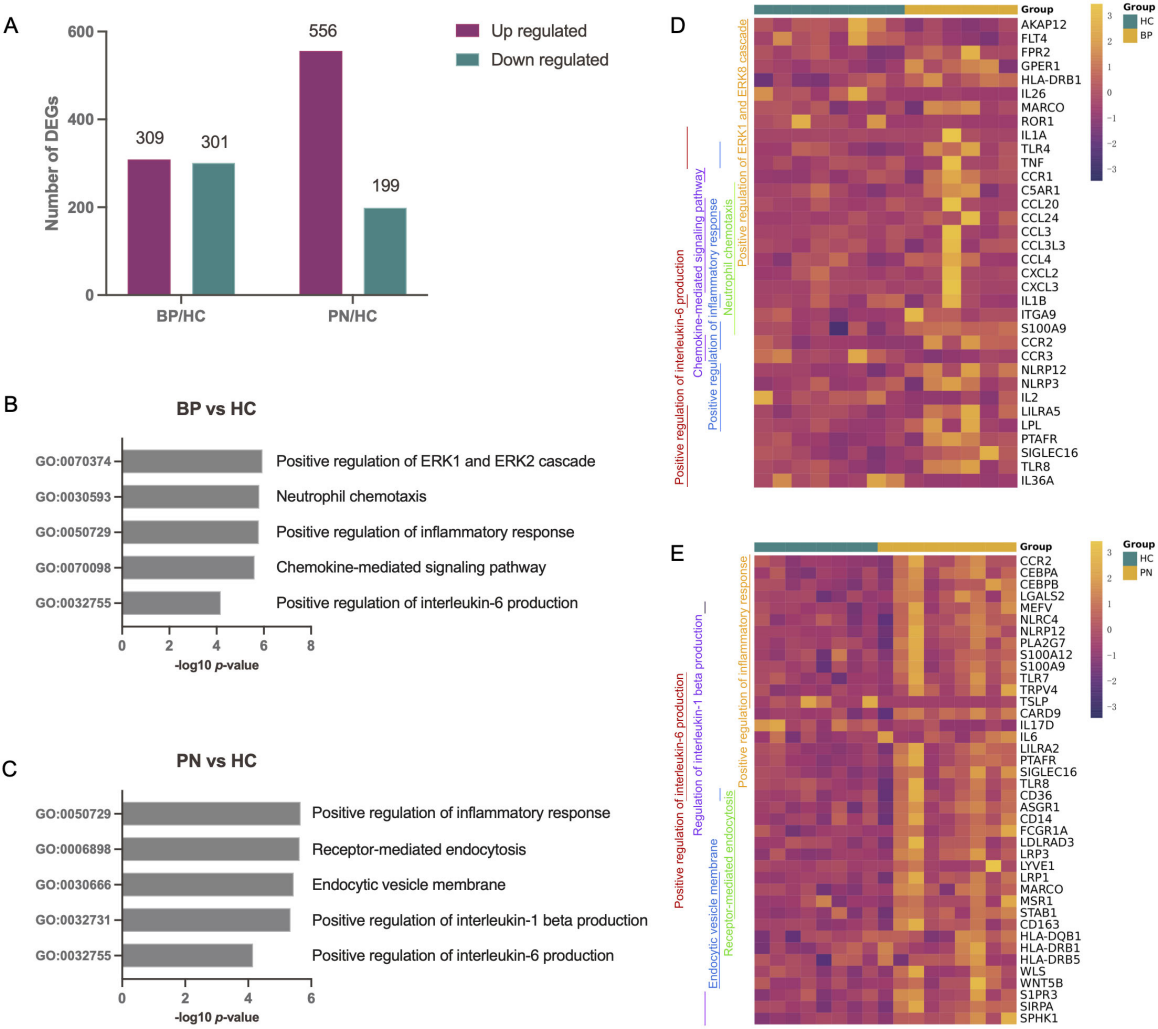
Differential expression analysis and pathway enrichment annotation of BP and PN. **(A)** Bar charts showing the number of DEGs in BP patients or PN patients in comparison to health controls. **(B, C)** Using Metascape, we identified five pathways that were enriched in the BP patients **(B)** or PN patients **(C)**. DEGs with an absolute value of log2-fold change greater than 1 (|log2FC| > 1) were used for the pathway analysis. **(D, E)** The genes corresponding to the GO pathways were shown on the heatmaps. Each GO pathway is represented by the colored bar on the left.

From two participant groups, skin lesions and PBMC samples were collected. The discovery group had 6 BP patients, 9 PN patients, and 8 HCs, with PBMC samples subjected to RNA-seq for DEG identification. The validation cohort consisted of 7 BP patients, 10 PN patients, and 5 HCs, provided clinical samples to validate DEGs (**Table 1**). All patients met established disease criteria and exhibited characteristic symptoms, such as high pruritus-numeric rating scale (P-NRS) scores, or eosinophil counts (EOS) levels. RNA-seq of PBMC samples identified DEGs relative to HCs (**Supplementary Figures 1A, B**). In BP patients, 610 DEGs were identified, including 309 upregulated and 301 downregulated genes; in PN patients, 755 DEGs were identified with 556 upregulated and 199 downregulated genes (**Figure 1A**). GO analysis of DEGs using Metascape revealed significant enrichment in inflammatory and IL-6 pathways for both diseases (**Figures 1B, C**), hinting at their key roles in BP and PN pathogenesis. **Figures 1D, E** showed enriched pathways and expression levels, potentially revealing therapeutic targets and key regulators in BP and PN inflammation or pruritus.

### 3.2 Functional annotation of common DEGs between BP and PN

**Figure 2:**
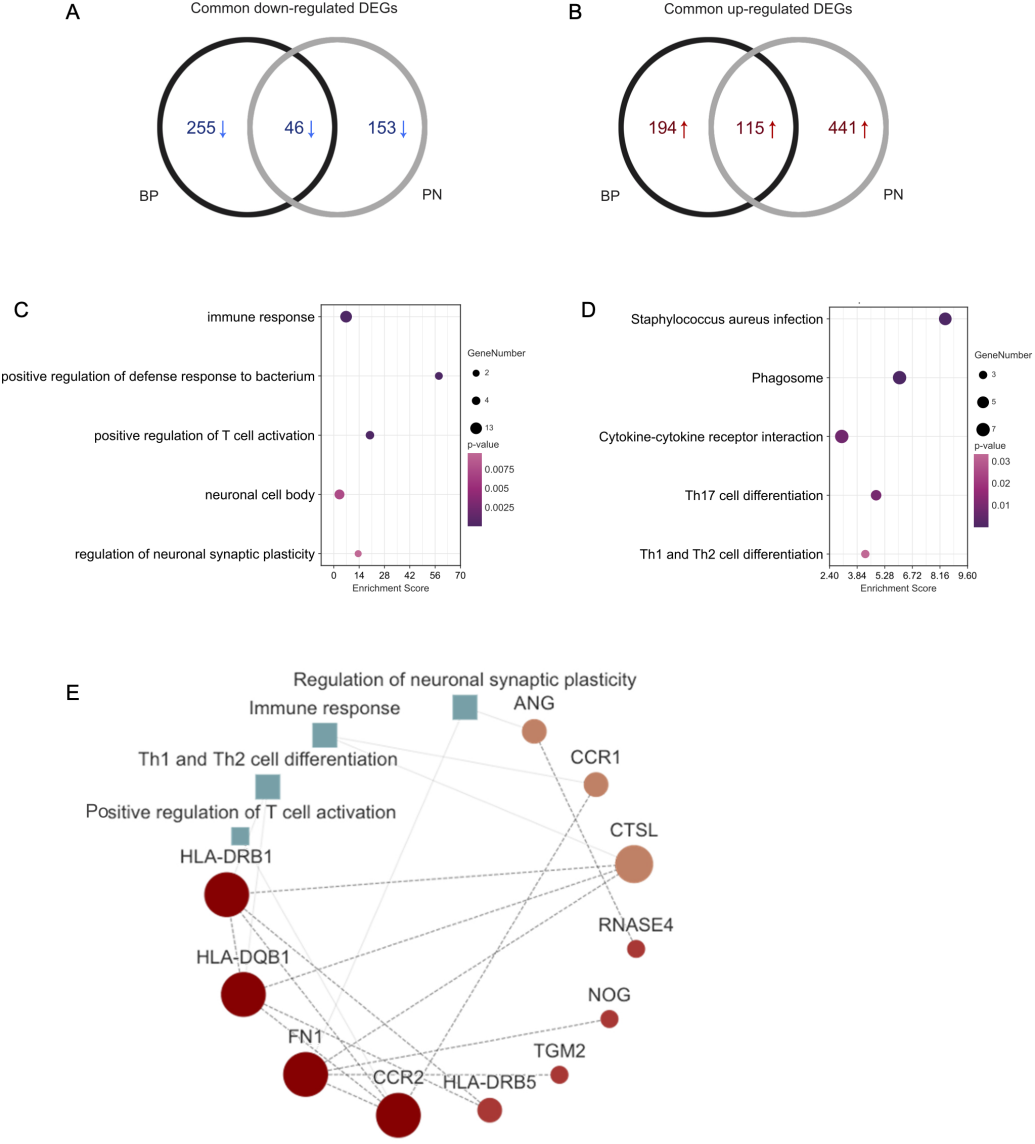
Functional annotation of DEGs. **(A)** The Venn diagram shows the intersection of downregulated DEGs from the BP and PN samples, respectively. **(B)** The Venn diagram shows the intersection of upregulated DEGs obtained from the BP and PN cohorts, respectively. **(C)** Bubble chart illustrating the significant enrichment terms of co-expressed DEGs in terms of GO enrichment analysis. **(D)** Bubble chart illustrating the significant enrichment terms of co-expressed DEGs in the KEGG analysis. **(E)** The PPI networks including the interactions of DEGs, and positive regulation of T-cell activation, regulation of neuronal synaptic plasticity, immune response, and Th1 and Th2 cell differentiation.

Using an online Venn diagram tool, we identified 161 DEGs common to BP and PN, with 46 downregulated and 115 upregulated (**Figures 2A, B**). To explore their functions roles, GO and KEGG analyses were performed. GO analysis revealed that these common DEGs were closely associated with neural and immune pathways, encompassing immune response, positive regulation of defense response to bacteria, positive regulation of T-cell activation, neuronal cell body, and neuronal synaptic plasticity (**Figure 2C**). KEGG analysis indicated that these DEGs were primarily enriched in pathways such as Staphylococcus aureus infection, phagosome, cytokine-cytokine receptor interaction, Th17 cell differentiation, and Th1 and Th2 cell differentiation (**Figure 2D**). Subsequently, a PPI network was constructed to examine the interactions among the DEGs. The PPI network revealed clusters tied to processes such as positive regulation of T-cell activation, regulation of neuronal synaptic plasticity, immune response, and Th1 and Th2 cell differentiation (**Figure 2E**).

### 3.3 Identification of key genes involved in the pathogenesis of BP and PN

**Figure 3:**
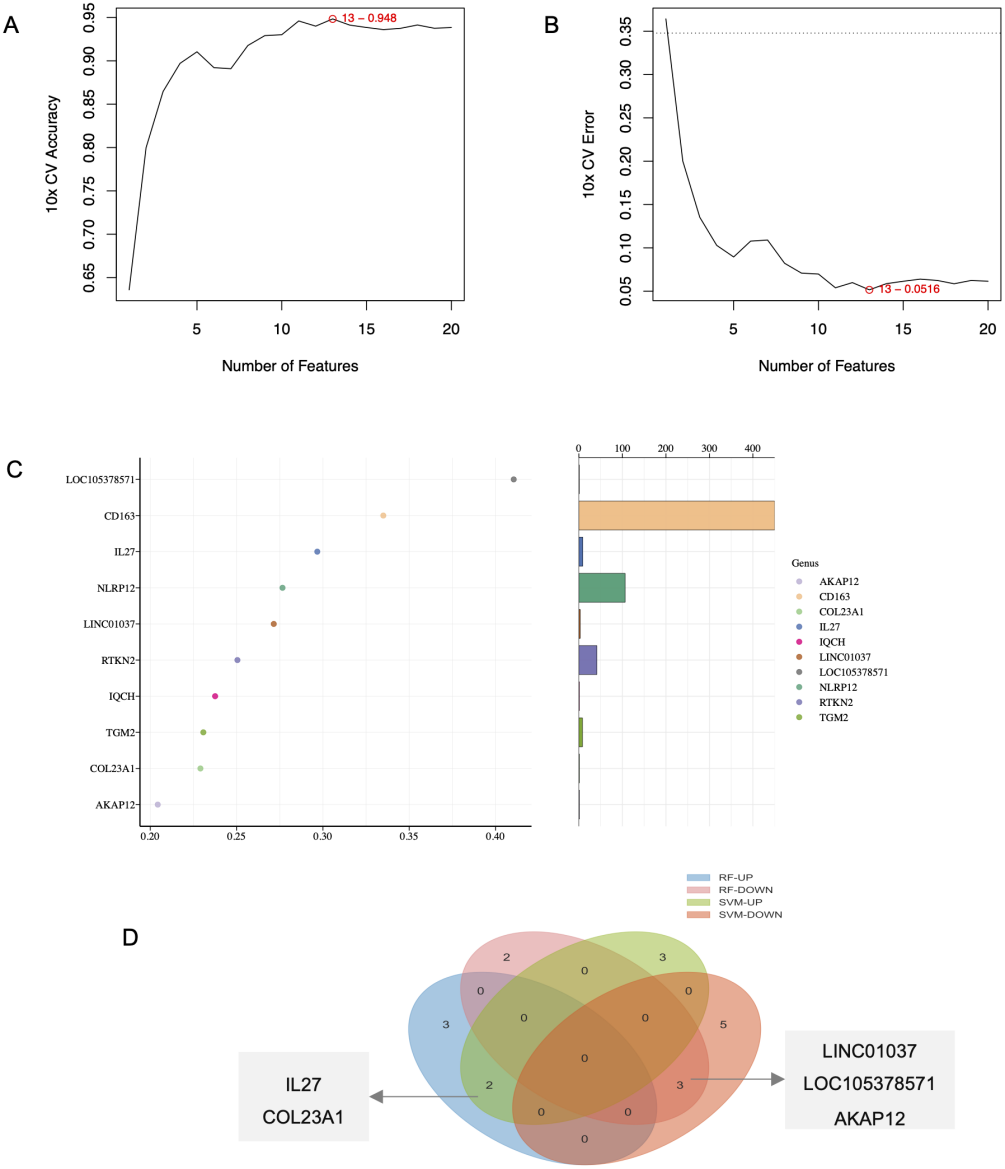
Identification of key genes in BP and PN. **(A, B)** Identification of hub genes for SVM algorithms. **(C)** Identification of hub genes for RF algorithms. **(D)** Venn diagram screening overlapping hub genes from SVM and RF.

We applied SVM and RF algorithms to identify shared core genes between BP and PN. The SVM algorithm selected key genes from BP-PN gene expression data (**Supplementary Table 1**), maximizing classification accuracy and predictive performance at the optimal feature count (**Figures 3A, B**). The RF algorithm identified another set of crucial genes, including AKAP12, CD163, COL23A1, IL-27, IQCH, LINC01037, LOC105378571, etc. (**Figure 3C**). To validate essential core genes, overlapping results from both algorithms were analyzed using a Venn diagram. IL-27, COL23A1, LINC01037, LOC105378571, and AKAP12 emerged as highly recognized core genes by both SVM and RF (**Figure 3D**).

### 3.4 Validation of key genes involved in the pathogenesis of BP and PN

**Figure 4:**
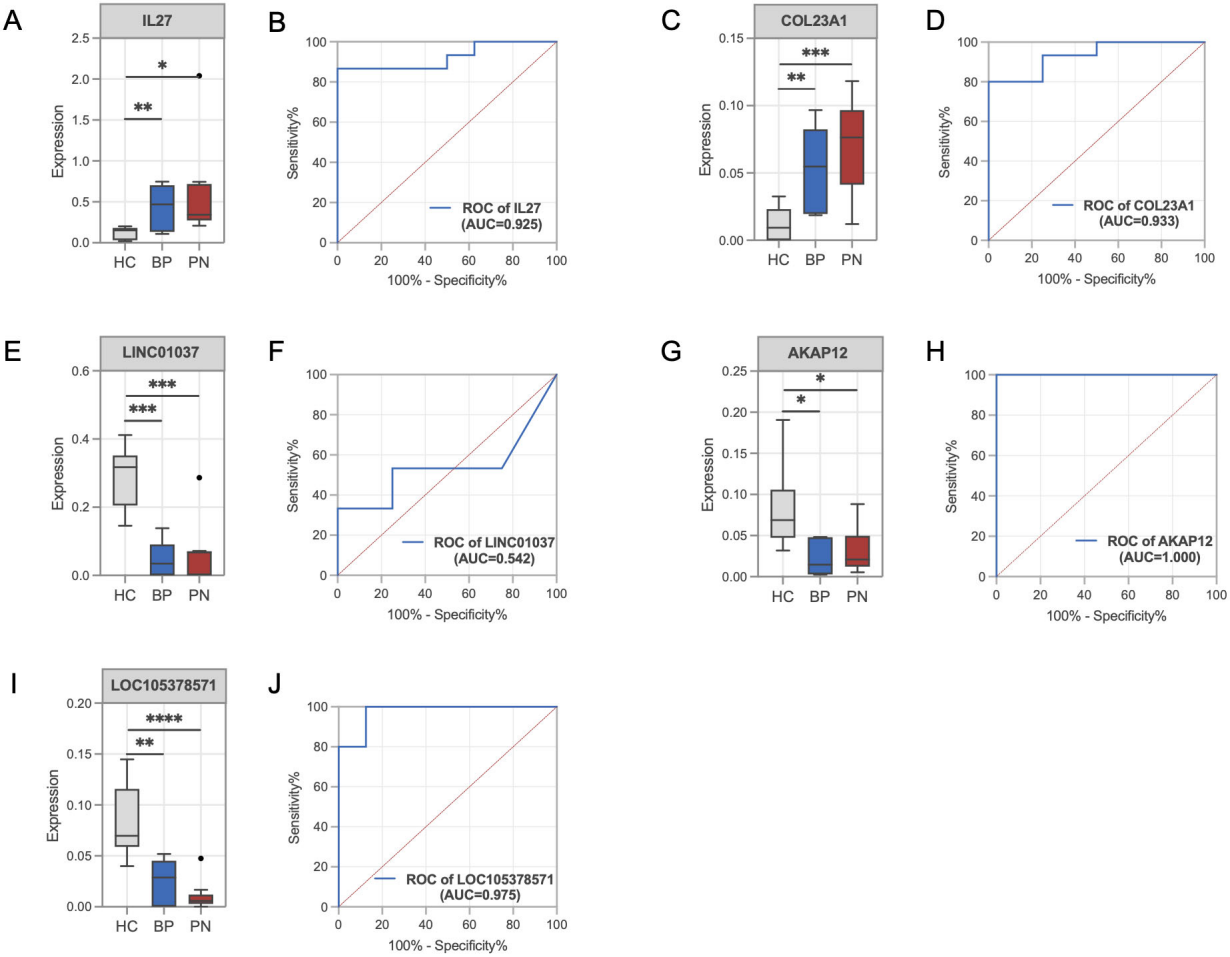
Validation of the key genes in BP and PN. **(A)** Histograms showing the expression levels of IL-27 in the gene profile of BP and PN samples compared to healthy controls. **(B)** ROC curve analysis of the IL-27. **(C)** Histograms showing the expression levels of COL23A1 in the gene profile of BP and PN samples compared to healthy controls. **(D)** ROC curve analysis of the COL23A1. **(E)** Histograms showing the expression levels of LINC01037 in the gene profile of BP and PN samples compared to healthy controls. **(F)** ROC curve analysis of the LINC01037. **(G)** Histograms showing the expression levels of AKAP12 in the gene profile of BP and PN samples compared to healthy controls. **(H)** ROC curve analysis of the AKAP12. **(I)** Histograms showing the expression levels of LOC105378571 in the gene profile of BP and PN samples compared to healthy controls. **(J)** ROC curve analysis of the LOC105378571. * *P* < 0.05, ** *P* < 0.01, *** *P* < 0.001, **** *P* < 0.0001.

Next, we validated the expression changes of key genes in BP and PN samples. Compared with the HC group, the gene expression levels of IL-27 (**Figure 4A**) and COL23A1 (**Figure 4C**) were significantly upregulated in both BP and PN, while the expression levels of LINC01037 (**Figure 4E**), AKAP12 (**Figure 4G**), and LOC105378571 (**Figure 4I**) were significantly downregulated. ROC curve analysis showed that IL-27 (**Figure 4B**, AUC=0.925), COL23A1 (**Figure 4D**, AUC=0.933), AKAP12 (**Figure 4H**, AUC=1.000), and LOC105378571 (**Figure 4J**, AUC=0.975) achieved high areas under the curve, indicating robust robust diagnostic and predictive capabilities. In contrast, LINC01037 (**Figure 4F**, AUC=0.542) exhibited a lower AUC value.

### 3.5 Identification of the association between key genes and clinical features

**Figure 5:**
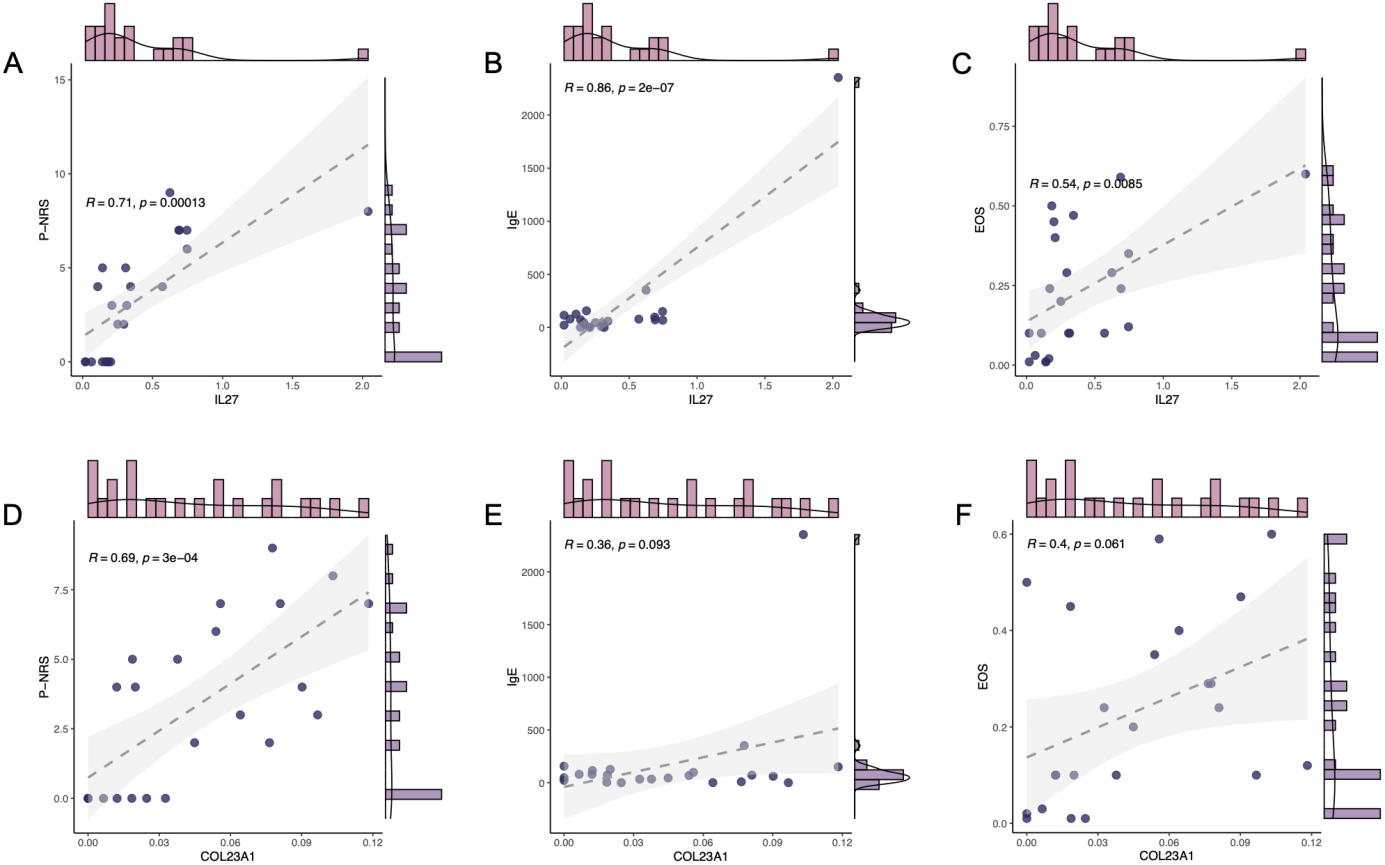
Clinical characteristics of key gene. The correlation between mRNA expression level of IL-27 and the clinical characteristics, P-NRS **(A)**, IgE **(B)**, and EOS **(C)**. The correlation between mRNA expression level of COL23A1 and the clinical characteristics, P-NRS **(D)**, IgE **(E)**, and EOS **(F)**.

We delved into the key genes IL-27 and COL23A1, which were significantly upregulated in both BP and PN diseases, to ascertain their roles. By analyzing patient clinical samples and the scratching-related metrics P-NRS, immunoglobulin E (IgE) levels, and EOS, we found strong positive correlations between IL-27 mRNA expression and P-NRS (R = 0.71, *P* = 0.00013) (**Figure 5A**), IgE (R = 0.86, *P* = 2e-07) (**Figure 5B**), suggesting its potential as a crucial indicator of itching perception. A moderate correlation with EOS (R = 0.54, *P* = 0.0085) (**Figure 5C**) was also observed. In contrast, COL23A1 showed weaker associations with P-NRS (R = 0.69, *P* = 3e-04) (**Figure 5D**) and insignificant correlations with IgE (R = 0.36, *P* = 0.093) and EOS (R = 0.4, *P* = 0.061) (**Figures 5E, F**). Based on these findings, we hypothesized that IL-27 plays a pivotal role in regulating pruritus in regulating pruritus in BP and PN.

### 3.6 Verification and detection of IL-27 expression levels in validation clinical samples of BP and PN

**Figure 6:**
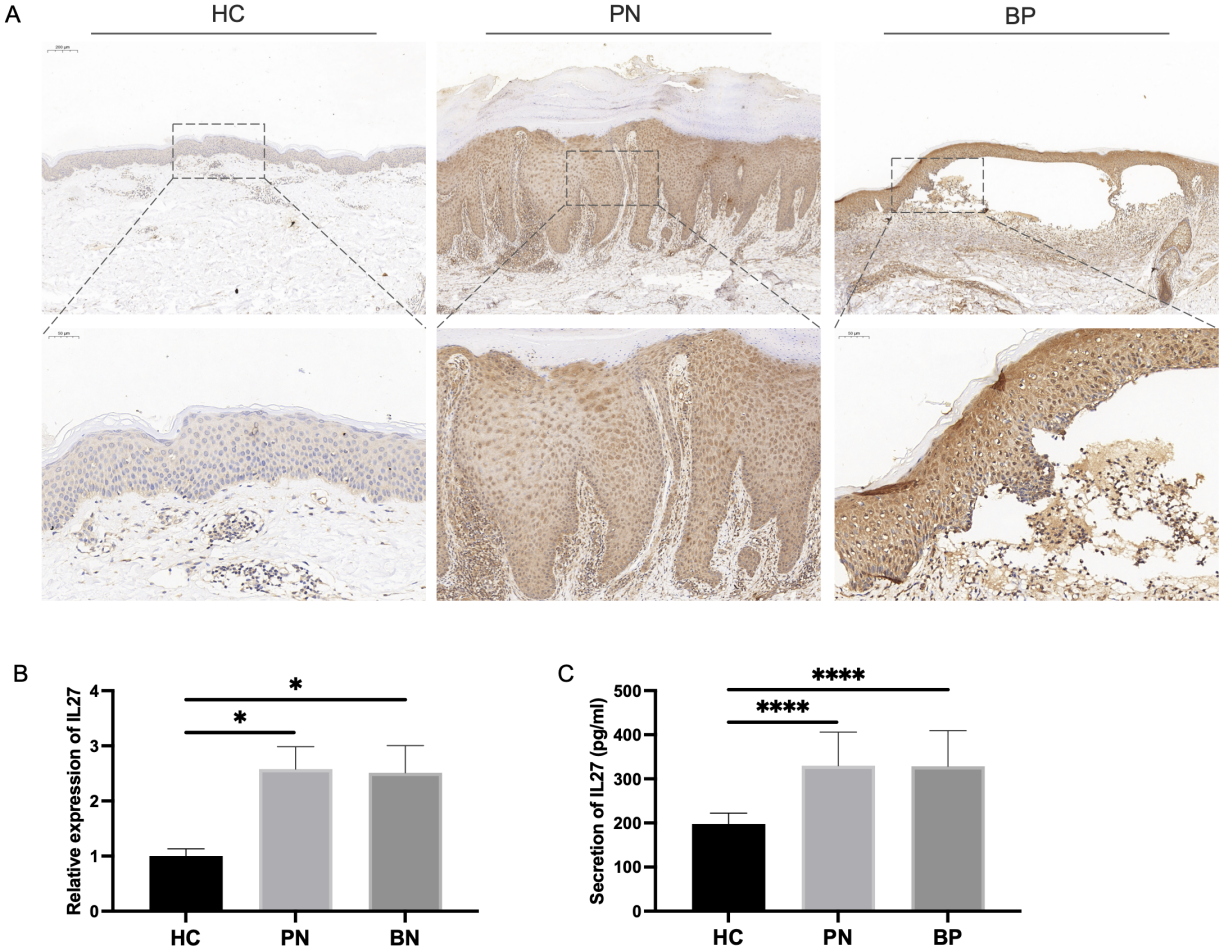
Verification of IL-27 expression in validation clinical samples. **(A)** IHC staining for IL-27 expression in clinical BP (N=7), PN (N=10) and HC (N=5) lesions. **(B)** qPCR analysis showing the expression level of IL-27 expression in clinical PBMCs. **(C)** ELISA detecting the serum secretion of IL-27. * *P* < 0.05, **** *P* < 0.0001.

To further investigate the potential correlation between IL-27 and pruritus, as well as validate the diagnostic value of altered IL-27 expression in BP and PN, we collected clinical samples and systematically analyzed the expression of IL-27. IHC staining of the affected skin lesion areas revealed strong positive expression of IL-27 in the lesion tissues of BP and PN patients compared to HCs (**Figure 6A**). qPCR analysis showed significantly higher IL-27 mRNA expression levels in PBMCs from BP and PN patients compared to the HC group (**Figure 6B**). Furthermore, ELISA results from serum samples indicated that the concentrations of IL-27 in the sera of BP and PN patients were also significantly higher than those in the HC group (**Figure 6C**). These findings underscore the crucial role of IL-27 in the pathogenesis of BP and PN and its potential as a diagnostic biomarker.

### 3.7 GSEA analysis in BP and PN

**Figure 7:**
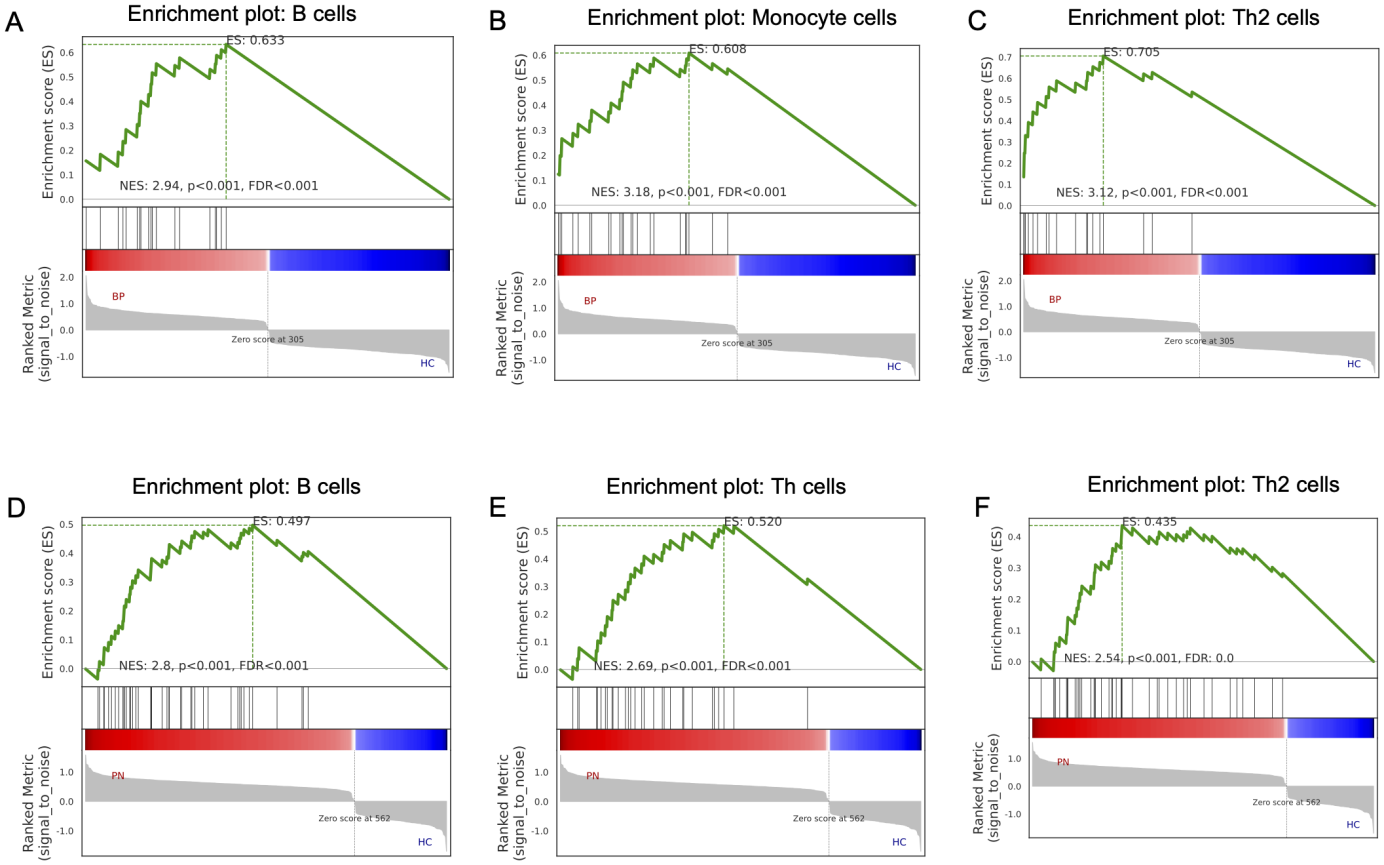
GSEA analysis in the BP and PN. GSEA analysis revealed the enriched cells in the samples of the BP dataset: **(A)** B cells, **(B)** Monocyte cells, **(C)** Th2 cells. GSEA analysis revealed the enriched cells in the samples of the PN dataset: **(D)** B cells, **(E)** Th cells, **(F)** Th2 cells.

Furthermore, we utilized GSEA to assess the immune cell types and states in BP and PN sample datasets. The results indicated that the most significantly upregulated immune infiltration terms in BP were mainly concentrated in pathways related to B cells (**Figure 7A**), monocytes (**Figure 7B**), and Th2 cells (**Figure 7C**). And PN samples exhibited B cell (**Figure 7D**), Th cell (**Figure 7E**), and Th2 cell pathway infiltration (**Figure 7F**). The similarities in immune cell types between BP and PN further suggest potential overlaps in the pathogenesis of these two diseases.

## 4 Discussion

BP and PN are chronic, pruritic Type 2 skin diseases with limited research on specific serum biomarkers. This study, pioneeringly, sequenced PBMCs from patients with BP and PN, alongside HCs, to analyze transcriptome changes linked to pruritus. We innovatively identified IL-27 as a novel potential biomarker, correlated with pruritus and inflammation severity. This finding paves the way for new treatments and deepens our knowledge of IL-27 in BP and PN.

Previous studies have demonstrated that Type 2 immune responses and key cytokines, such as IL-4, IL-13, and IL-31 play crucial roles in the pathophysiological process of chronic pruritus (30). Although BP and PN differ in clinical manifestations, they may share common pathophysiological processes. Persistent pruritus in BP is largely driven by robust Th2 cell-mediated immune responses, characterized by elevated Th2 lymphocyte subsets and mast cell infiltration in the lesions, along with elevated EOS counts and IgE levels in the peripheral blood (31–34). Notably, EOS are the primary source of itch-inducing cytokines in the skin and serum of BP patients (35). In PN, the upregulation of IL-4 and IL-13 sustains and amplifies the Th2 cell-mediated immune response, further recruiting EOS and mast cells, stimulating IgE production, and exacerbating the vicious cycle of itching through interactions with IL-31 (36–39) These cytokines not only act directly as pruritogens for primary sensory neurons and modify sensitivity to other itch mediators, but also mediate peripheral sensitization mechanisms as well as abnormal neuro-immune-epithelial interactions (40–42). Our sequencing results indicate shared pathways in BP and PN pathogenesis, including the positive regulation of T-cell activation, Th2 cell differentiation, and modulation of neuronal synaptic plasticity. The findings suggest that, as typical Type 2 inflammatory skin diseases, BP and PN may involve significant neuro-immune interactions that play a crucial role in the initiation and maintenance of pruritus. Additionally, these pathological changes related to inflammation and itch perception are also prominent in the peripheral circulation.

By employing the SVM and RF machine learning strategies, we discovered IL-27 as a key gene highly expressed in the PBMCs of patients with BP and PN. Correlation analysis of clinical data revealed a strong positive correlation between IL-27 expression and clinical P-NRS, as well as significant correlations with the allergic maker IgE levels and EOS counts. IL-27, a member of the IL-6/IL-12 cytokine family, is primarily produced by myeloid cells and signals through a receptor composed of IL-27Rα and gp130 subunits, that is present not only on innate immune cells but also on adaptive immune cells, allowing IL-27 to broadly influence immune responses (43, 44).

In the skin microenvironment, IL-27 is primarily secreted by epidermal keratinocytes and antigen-presenting cells, such as macrophages and dendritic cells (45). IL-27 exerts a regulatory effect on Th2 cells, and has been shown to maintain inflammatory responses in chronic eczema by inducing the production of C-X-C motif chemokine ligand 10 and enhancing the survival of epidermal cells (46). Suwanpradid et al. found that IL-27 derived from macrophages can promote skin hypersensitivity and play a role in human allergic contact dermatitis (47). Xu WD et al. showed that IL-27 regulate eosinophil function by stimulating downstream signaling, such as nuclear factor-kappa B and mitogen-activated protein kinase pathways (48). Additionally, IL-27 has been implicated in cutaneous inflammation and pruritus, as it can upregulate the transcription of protease-activated receptor 2 in the skin, which in turn enhances neural fiber density, elevates the expression of nerve growth factor and endothelin-1, intensifies hypersensitivity reactions, and potentiates the responsiveness of dorsal root ganglion cells to non-histaminergic pruritogens, thereby inducing skin inflammation and itching.

Moreover, GSEA revealed notable similarities in B-cell and Th-cell states between BP and PN, also indicating shared immunopathogenesis. Elevated IL-27 levels in PBMCs, skin lesions, and serum of both patients suggests its involvement in immune cell activation and neural hypersensitivity. Given its immunoregulatory properties, IL-27 has been considered a potential therapeutic target for Th2-mediated allergic diseases. Our current findings suggest the importance of IL-27 in peripheral itch signal transmission and may be an important pruritus feature in BP and PN.

This study is the first to directly sequence PBMCs from BP and PN patients, using proprietary clinical samples to mitigate biases associated with database reliance on public datasets. By integrating bioinformatics and machine learning, we pinpointed IL-27 as a critical player in BP and PN pathogenesis, providing new insights into pruritus mechanisms. Yet, the small sample size limits the generalization of our findings. Larger-scale experiments and clinical studies are necessary to validate IL-27’s functions. We aim to overcome these limitations through further validations and analyses.

## 5 Conclusion

In summary, BP and PN share similar inflammatory and immunological features in their pathological processes, particularly in the mechanisms that trigger itching. This study is the first to propose and validate IL-27 as the common and specific key molecule linking these two pruritic inflammatory skin diseases. These findings not only provide novel insights into the potential relationship between BP and PN, but also opens new avenues for exploring more effective therapeutic strategies and developing novel therapeutic targets.

## Data Availability

The datasets presented in this study can be found in online repositories. The names of the repository/repositories and accession number(s) can be found in the article/**Supplementary Material**.

## Supplementary material

Supplementary Figure 1
**(A)** Volcano plot showing DEGs between BP and control groups. Green plots represent downregulated genes and red plots represent upregulated genes. **(B)** Volcano plot showing DEGs between PN and control groups.

## References

[1] Yosipovitch G Rosen JD Hashimoto T. Itch: From mechanism to (novel) therapeutic approaches. J Allergy Clin Immunol. (2018) 142:1375–90. doi: 10.1016/j.jaci.2018.09.005 30409247

[2] Butler DC Berger T Elmariah S Kim B Chisolm S Kwatra SG. Chronic pruritus: A review. JAMA. (2024) 331:2114–24. doi: 10.1001/jama.2024.4899 38809527

[3] Garcovich S Maurelli M Gisondi P Peris K Yosipovitch G Girolomoni G. Pruritus as a distinctive feature of type 2 inflammation. Vaccines (Basel). (2021) 9:303. doi: 10.3390/vaccines9030303 33807098 PMC8005108

[4] Kumar M Choi YG Wong T Li PH Chow BKC. Beyond the classic players: Mas-related G protein-coupled receptor member X2 role in pruritus and skin diseases. J Eur Acad Dermatol Venereol. (2024) 00: 1–11. doi: 10.1111/jdv.20249 PMC11851267 39044547

[5] Gomułka K Tota M Laska J Gojny K Sędek Ł. Serum concentration of IL-5 receptor (IL-5R) and associations with disease severity in patients with Chronic Spontaneous Urticaria (CSU) and Atopic Dermatitis (AD). Int J Mol Sci. (2024) 25:7598. doi: 10.3390/ijms25147598 39062845 PMC11276824

[6] Oh JS Seong GS Kim YD Choung SY. Deacetylasperulosidic acid ameliorates pruritus, immune imbalance, and skin barrier dysfunction in 2,4-dinitrochlorobenzene-induced atopic dermatitis NC/nga mice. Int J Mol Sci. (2021) 23:226. doi: 10.3390/ijms23010226 35008651 PMC8745491

[7] Li Y Chen W Zhu X Mei H Steinhoff M Buddenkotte J. Neuronal BST2: A pruritic mediator alongside protease-activated receptor 2 in the IL-27-driven itch pathway. J Invest Dermatol. (2024) 144(8):1829–42.e4. doi: 10.1016/j.jid.2024.01.025 38360199

[8] Steinhoff M Ahmad F Pandey A Datsi A AlHammadi A Al-Khawaga S. Neuroimmune communication regulating pruritus in atopic dermatitis. J Allergy Clin Immunol. (2022) 149(6):1875–98. doi: 10.1016/j.jaci.2022.03.010 35337846

[9] Wilson SR Thé L Batia LM Beattie K Katibah GE McClain SP. The epithelial cell-derived atopic dermatitis cytokine TSLP activates neurons to induce itch. Cell. (2013) 155(2):285–95. doi: 10.1016/j.cell.2013.08.057 PMC4041105 24094650

[10] Buhl T Ikoma A Kempkes C Cevikbas F Sulk M Buddenkotte J. Protease-activated receptor-2 regulates neuro-epidermal communication in atopic dermatitis. Front Immunol. (2020) 11:1740. doi: 10.3389/fimmu.2020.01740 32903402 PMC7435019

[11] Nattkemper LA Tey HL Valdes-Rodriguez R Lee H Mollanazar NK Albornoz C. The genetics of chronic itch: gene expression in the skin of patients with atopic dermatitis and psoriasis with severe itch. J Invest Dermatol. (2018) 138(6):1311–7. doi: 10.1016/j.jid.2017.12.029 29317264

[12] Moro F Fania L Sinagra JLM Salemme A Di Zenzo G. Bullous pemphigoid: trigger and predisposing factors. Biomolecules. (2020) 10:1432. doi: 10.3390/biom10101432 33050407 PMC7600534

[13] Hammers CM Stanley JR. Mechanisms of disease: pemphigus and bullous pemphigoid. Annu Rev Pathol. (2016) 11:175–97. doi: 10.1146/annurev-pathol-012615-044313 PMC5560122 26907530

[14] Schmidt E Zillikens D. Pemphigoid diseases. Lancet. (2013) 381:320–32. doi: 10.1016/S0140-6736(12)61140-4 23237497

[15] Williams KA Huang AH Belzberg M Kwatra SG. Prurigo nodularis: Pathogenesis and management. J Am Acad Dermatol. (2020) 83:1567–75. doi: 10.1016/j.jaad.2020.04.182 32461078

[16] Sutaria N Adawi W Goldberg R Roh YS Choi J Kwatra SG. Itch: pathogenesis and treatment. J Am Acad Dermatol. (2022) 86(1):17–34. doi: 10.1016/j.jaad.2021.07.078 34648873

[17] Liu T Wang Z Xue X Wang Z Zhang Y Mi Z. Single-cell transcriptomics analysis of bullous pemphigoid unveils immune-stromal crosstalk in type 2 inflammatory disease. Nat Commun. (2024) 15:5949. doi: 10.1038/s41467-024-50283-3 39009587 PMC11251189

[18] Tsoi LC Hacini-Rachinel F Fogel P Rousseau F Xing X Patrick MT. Transcriptomic characterization of prurigo nodularis and the therapeutic response to nemolizumab. J Allergy Clin Immunol. (2022) 149:1329–39. doi: 10.1016/j.jaci.2021.10.004 PMC8995330 34857395

[19] Hiraiwa T Matsumura N Mori T Kikuchi N Yamamoto T. Bullous pemphigoid developed after dramatic improvement of severe prurigo nodularis. Bras Dermatol. (2023) 98:689–91. doi: 10.1016/j.abd.2022.11.003 PMC10404485 37225629

[20] Sun M Chen ZR Ding HJ Feng J. Molecular and cellular mechanisms of itch sensation and the anti-itch drug targets. Acta Pharmacol Sin. doi: 10.1038/s41401-024-01400-x PMC11845708 39424975

[21] Du LX Zhu JY Mi WL. Cytokines and chemokines modulation of itch. Neuroscience. (2022) 495:74–85. doi: 10.1016/j.neuroscience.2022.05.035 35660453

[22] Oetjen LK Mack MR Feng J Whelan TM Niu H Guo CJ. Sensory neurons co-opt classical immune signaling pathways to mediate chronic itch. Cell. (2017) 171(1):217–28.e13. doi: 10.1016/j.cell.2017.08.006 PMC5658016 28890086

[23] Sofen H Bissonnette R Yosipovitch G Sun Z Engle SM Auxier AN. Efficacy and safety of vixarelimab, a human monoclonal oncostatin M receptor β antibody, in moderate-to-severe prurigo nodularis: a randomised, double-blind, placebo-controlled, phase 2a study. EClinicalMedicine. (2023) 57:101826. doi: 10.1016/j.eclinm.2023.101826 36816342 PMC9932343

[24] Dillon SR Sprecher C Hammond A Bilsborough J Rosenfeld-Franklin M Presnell SR. Interleukin 31, a cytokine produced by activated T cells, induces dermatitis in mice. Nat Immunol. (2004) 5(7):752–60. doi: 10.1038/ni1084 15184896

[25] Raap U Wichmann K Bruder M Ständer S Wedi B Kapp A. Correlation of IL-31 serum levels with severity of atopic dermatitis. J Allergy Clin Immunol. (2008) 122(2):421–3. doi: 10.1016/j.jaci.2008.05.047 18678344

[26] Silverberg JI Wollenberg A Reich A Thaçi D Legat FJ Papp KA. Nemolizumab with concomitant topical therapy in adolescents and adults with moderate-to-severe atopic dermatitis (ARCADIA 1 and ARCADIA 2): results from two replicate, double-blind, randomised controlled phase 3 trials. Lancet. (2024) 404(10451):445–60. doi: 10.1016/S0140-6736(24)01203-0 39067461

[27] Reich A. Nemolizumab: a new key player in the treatment of prurigo nodularis. Br J Dermatol. (2024) 191:154–5. doi: 10.1093/bjd/ljae189 38703061

[28] Borradori L Van Beek N Feliciani C Tedbirt B Antiga E Bergman R. Updated S2 K guidelines for the management of bullous pemphigoid initiated by the European Academy of Dermatology and Venereology (EADV). J Eur Acad Dermatol Venereol. (2022) 36(10):1689–704. doi: 10.1111/jdv.18220 35766904

[29] Elmariah S Kim B Berger T Chisolm S Kwatra SG Mollanazar N. Practical approaches for diagnosis and management of prurigo nodularis: United States expert panel consensus. J Am Acad Dermatol. (2021) 84(3):747–60. doi: 10.1016/j.jaad.2020.07.025 32682025

[30] Fassett MS Braz JM Castellanos CA Salvatierra JJ Sadeghi M Yu X. IL-31-dependent neurogenic inflammation restrains cutaneous type 2 immune response in allergic dermatitis. Sci Immunol. (2023) 8(88):eabi6887. doi: 10.1126/sciimmunol.abi6887 37831760 PMC10890830

[31] Messingham KN Crowe TP Fairley JA. The intersection of igE autoantibodies and eosinophilia in the pathogenesis of bullous pemphigoid. Front Immunol. (2019) 10:2331. doi: 10.3389/fimmu.2019.02331 31636640 PMC6787172

[32] Schulze FS Beckmann T Nimmerjahn F Ishiko A Collin M Köhl J. Fcγ receptors III and IV mediate tissue destruction in a novel adult mouse model of bullous pemphigoid. Am J Pathol. (2014) 184:2185–96. doi: 10.1016/j.ajpath.2014.05.007 25043618

[33] Verraes S Hornebeck W Polette M Borradori L Bernard P. Respective contribution of neutrophil elastase and matrix metalloproteinase 9 in the degradation of BP180 (type XVII collagen) in human bullous pemphigoid. J Invest Dermatol. (2001) 117:1091–6. doi: 10.1046/j.0022-202x.2001.01521.x 11710917

[34] Marzano AV Tedeschi A Berti E Fanoni D Crosti C Cugno M. Activation of coagulation in bullous pemphigoid and other eosinophil-related inflammatory skin diseases. Clin Exp Immunol. (2011) 165:44–50. doi: 10.1111/j.1365-2249.2011.04391.x 21488867 PMC3110320

[35] Webb LM Tait Wojno ED. The role of rare innate immune cells in Type 2 immune activation against parasitic helminths. Parasitology. (2017) 144:1288–301. doi: 10.1017/S0031182017000488 PMC5962964 28583216

[36] Ma F Gharaee-Kermani M Tsoi LC Plazyo O Chaskar P Harms P. Single-cell profiling of prurigo nodularis demonstrates immune-stromal crosstalk driving profibrotic responses and reversal with nemolizumab. J Allergy Clin Immunol. (2024) 153:146–60. doi: 10.1016/j.jaci.2023.07.005 PMC11231883 37506977

[37] Lloyd CM Snelgrove RJ. Type 2 immunity: Expanding our view. Sci Immunol. (2018) 3:eaat1604. doi: 10.1126/sciimmunol.aat1604 29980619

[38] Neis MM Peters B Dreuw A Wenzel J Bieber T Mauch C. Enhanced expression levels of IL-31 correlate with IL-4 and IL-13 in atopic and allergic contact dermatitis. J Allergy Clin Immunol. (2006) 118:930–7. doi: 10.1016/j.jaci.2006.07.015 17030248

[39] Dubin C Del Duca E Guttman-Yassky E. The IL-4, IL-13 and IL-31 pathways in atopic dermatitis. Expert Rev Clin Immunol. (2021) 17:835–52. doi: 10.1080/1744666X.2021.1940962 34106037

[40] Campion M Smith L Gatault S Métais C Buddenkotte J Steinhoff M. Interleukin-4 and interleukin-13 evoke scratching behaviour in mice. Exp Dermatol. (2019) 28:1501–4. doi: 10.1111/exd.14034 31505056

[41] Schmelz M. Itch and pain differences and commonalities. Handb Exp Pharmacol. (2015) 227:285–301. doi: 10.1007/978-3-662-46450-2_14 25846624

[42] Hawro T Saluja R Weller K Altrichter S Metz M Maurer M. Interleukin-31 does not induce immediate itch in atopic dermatitis patients and healthy controls after skin challenge. Allergy. (2014) 69:113–7. doi: 10.1111/all.12316 24251414

[43] Müller SI Friedl A Aschenbrenner I Esser-von Bieren J Zacharias M Devergne O. A folding switch regulates interleukin 27 biogenesis and secretion of its α-subunit as a cytokine. Proc Natl Acad Sci U S A. (2019) 116:1585–90. doi: 10.1073/pnas.1816698116 PMC6358668 30651310

[44] Pflanz S Hibbert L Mattson J Rosales R Vaisberg E Bazan JF. WSX-1 and glycoprotein 130 constitute a signal-transducing receptor for IL-27. J Immunol. (2004) 172:2225–31. doi: 10.4049/jimmunol.172.4.2225 14764690

[45] Yoshimoto T Yasuda K Mizuguchi J Nakanishi K. IL-27 suppresses Th2 cell development and Th2 cytokines production from polarized Th2 cells: a novel therapeutic way for Th2-mediated allergic inflammation. J Immunol. (2007) 179:4415–23. doi: 10.4049/jimmunol.179.7.4415 17878337

[46] Wittmann M Zeitvogel J Wang D Werfel T. IL-27 is expressed in chronic human eczematous skin lesions and stimulates human keratinocytes. J Allergy Clin Immunol. (2009) 124:81–9. doi: 10.1016/j.jaci.2009.04.026 19523673

[47] Suwanpradid J Lee MJ Hoang P Kwock J Floyd LP Smith JS. IL-27 derived from macrophages facilitates IL-15 production and T cell maintenance following allergic hypersensitivity responses. Front Immunol. (2021) 12:713304. doi: 10.3389/fimmu.2021.713304 34659203 PMC8515907

[48] Xu WD Wang DC Zhao M Huang AF. An updated advancement of bifunctional IL-27 in inflammatory autoimmune diseases. Front Immunol. (2024) 15:1366377. doi: 10.3389/fimmu.2024.1366377 38566992 PMC10985211

