# Supplementary figures and images for: IL-27 as a novel biomarker for pruritus in nodular prurigo and bullous pemphigoid
Source: Front Immunol. 2024 Dec 13;15:1499868. doi: 10.3389/fimmu.2024.1499868 (PMC11681427; doi:10.3389/fimmu.2024.1499868)

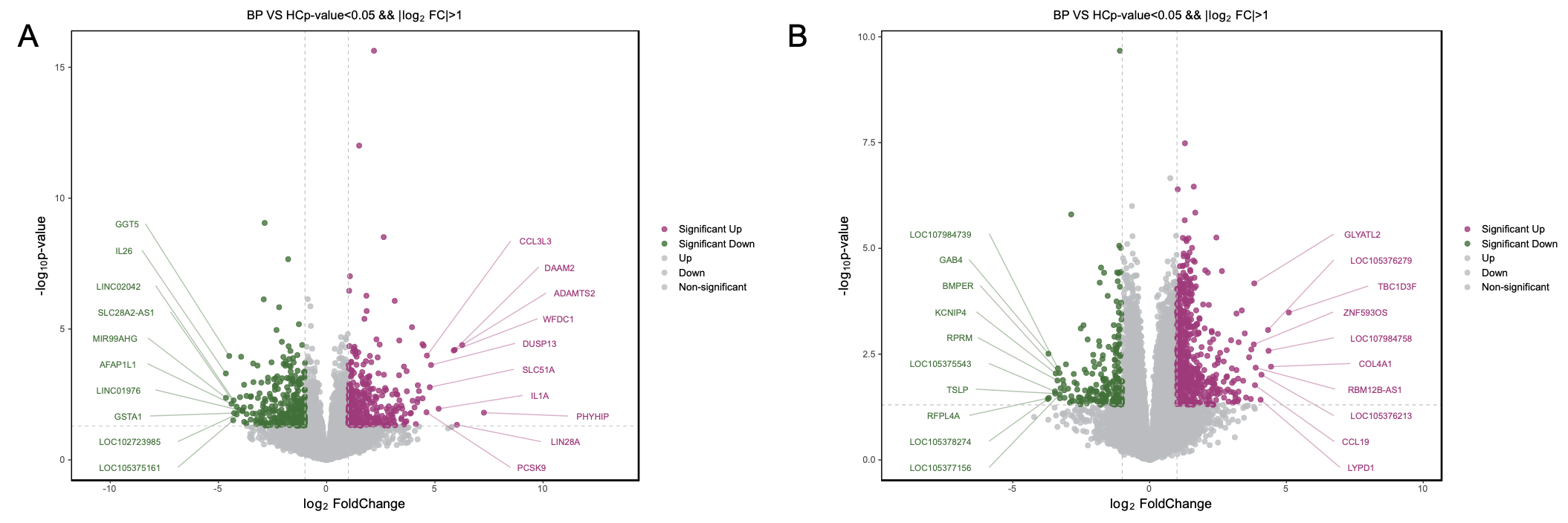

Supplement: Supplementary Figure 1 — (A) Volcano plot showing DEGs between BP and control groups. Green plots represent downregulated genes and red plots represent upregulated genes. (B) Volcano plot showing DEGs between PN and control groups. [file Image1.tiff]

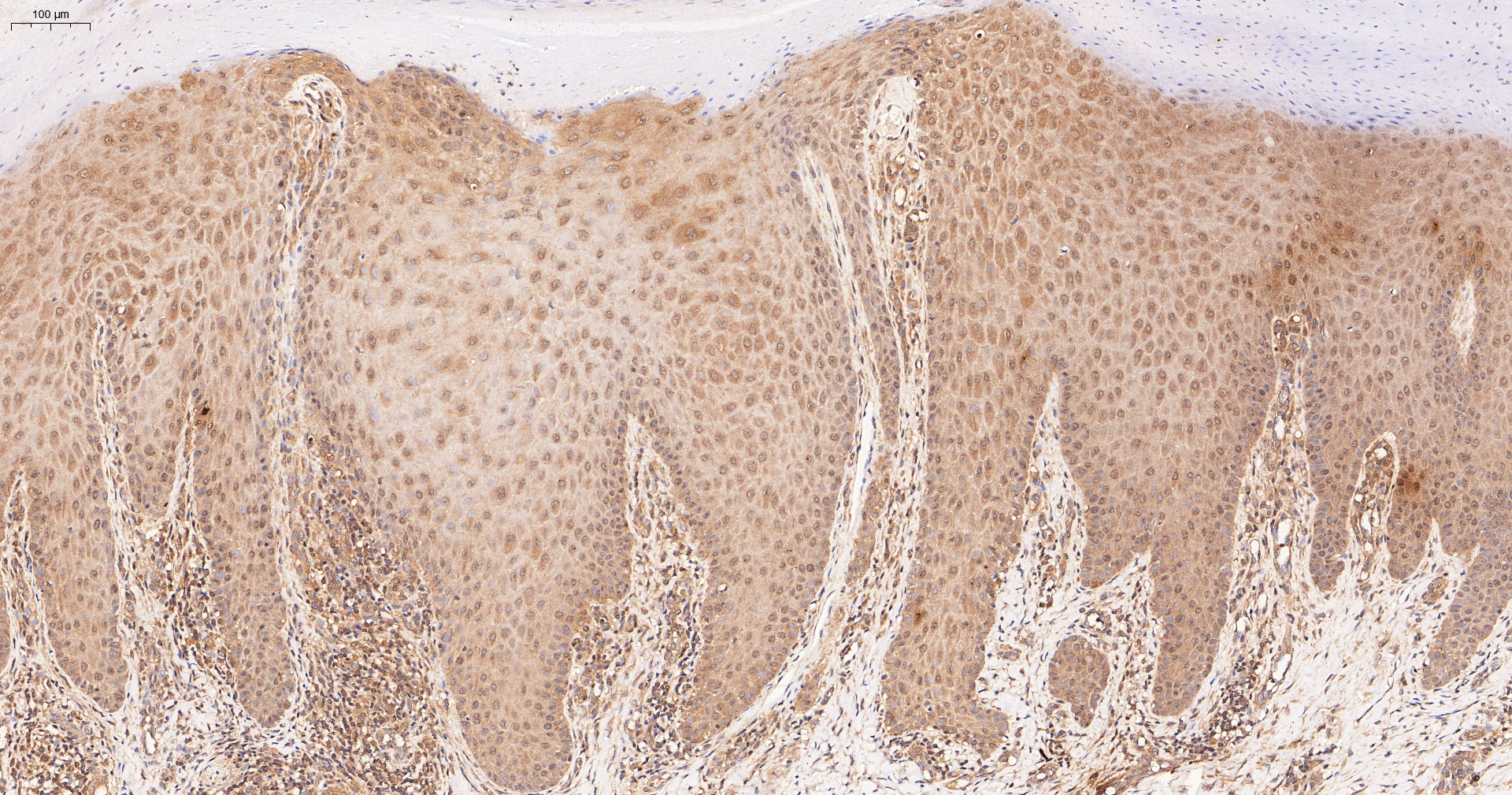

Supplement: Supplementary file 3 [file DataSheet1.zip › data/IHC/PN_1_10.0x.jpg]

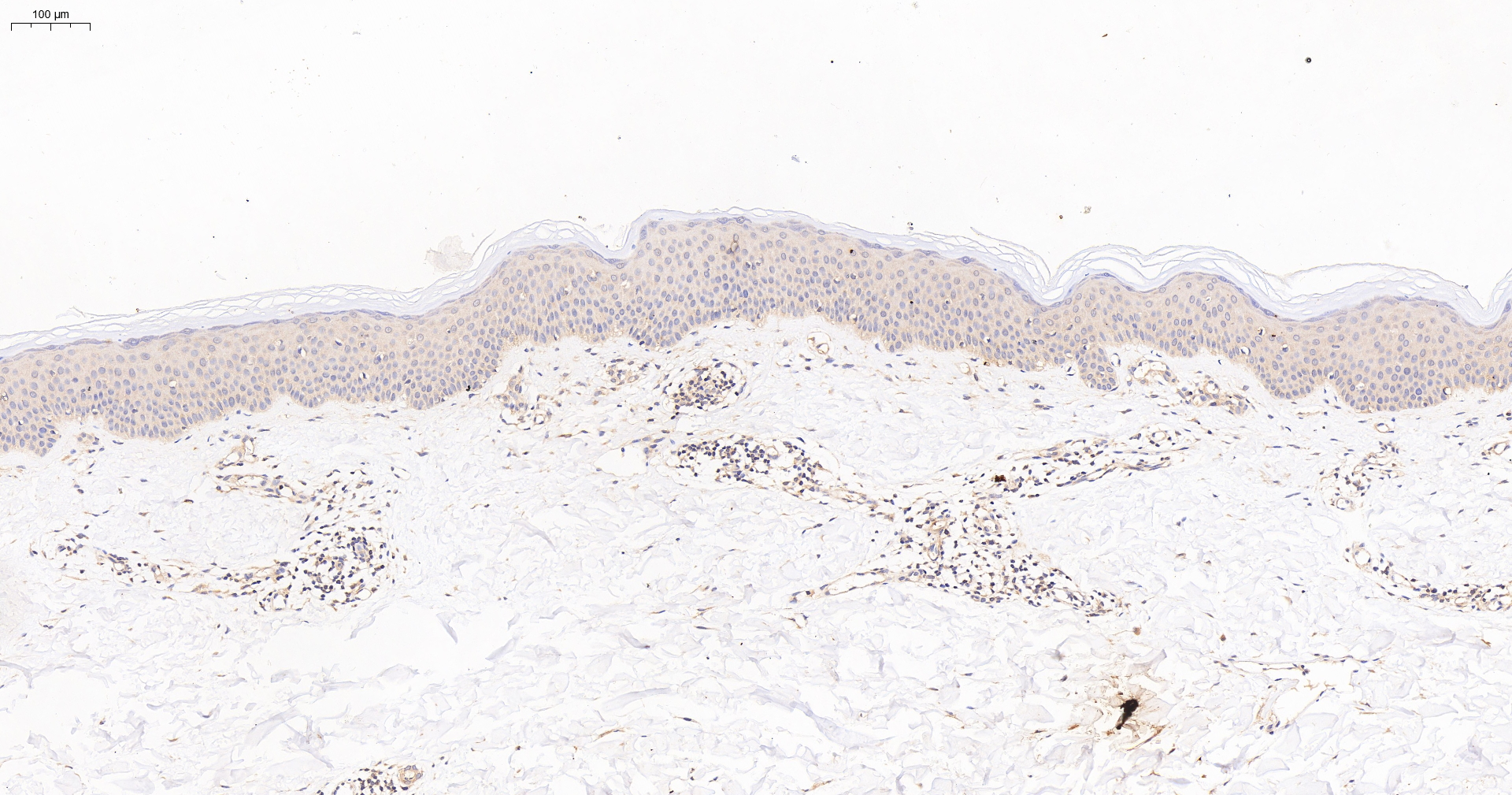

Supplement: Supplementary file 3 [file DataSheet1.zip › data/IHC/HC_1_10.jpg]

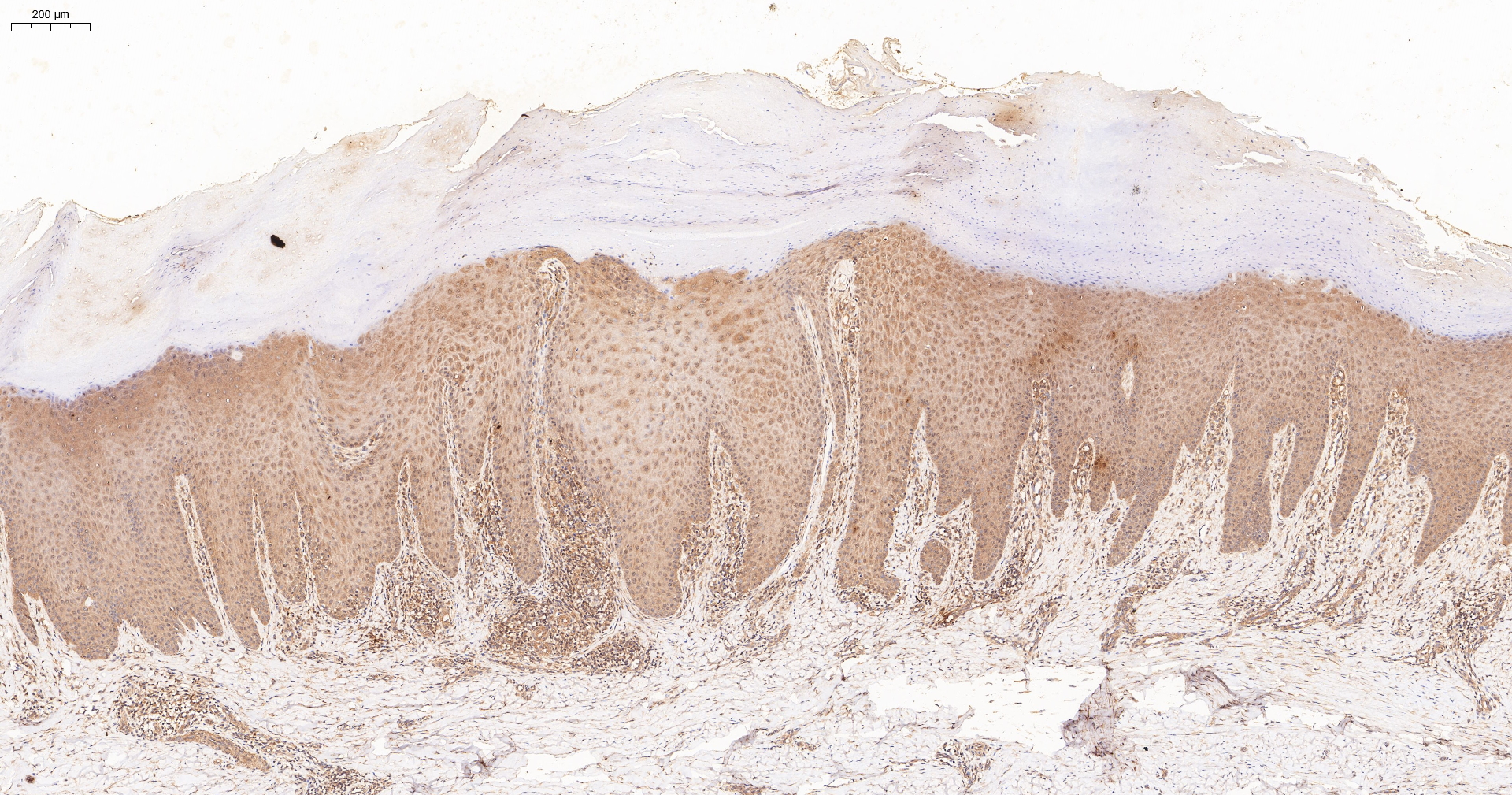

Supplement: Supplementary file 3 [file DataSheet1.zip › data/IHC/PN_1_5.0x.jpg]

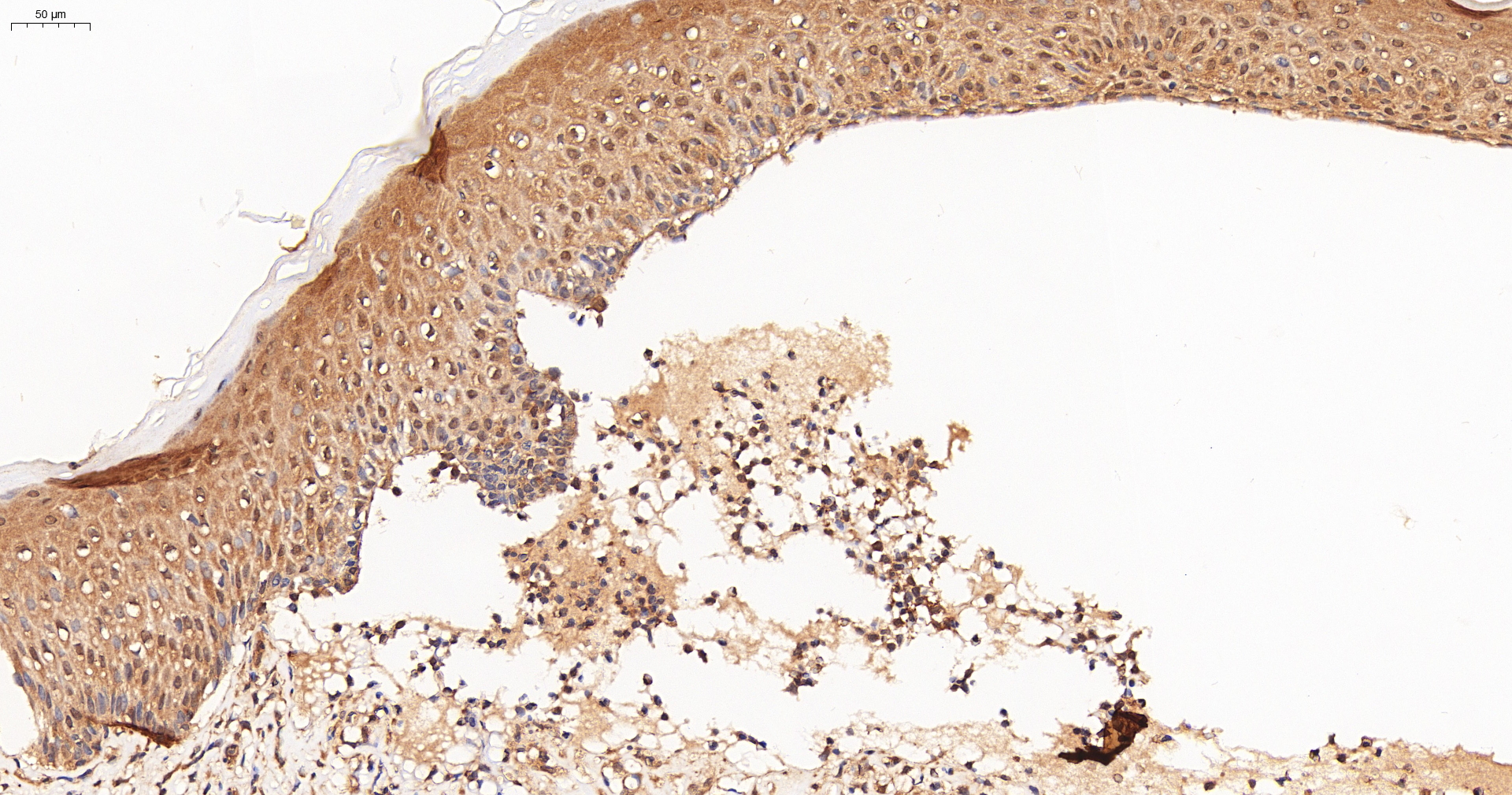

Supplement: Supplementary file 3 [file DataSheet1.zip › data/IHC/BP_1_20.0x.jpg]

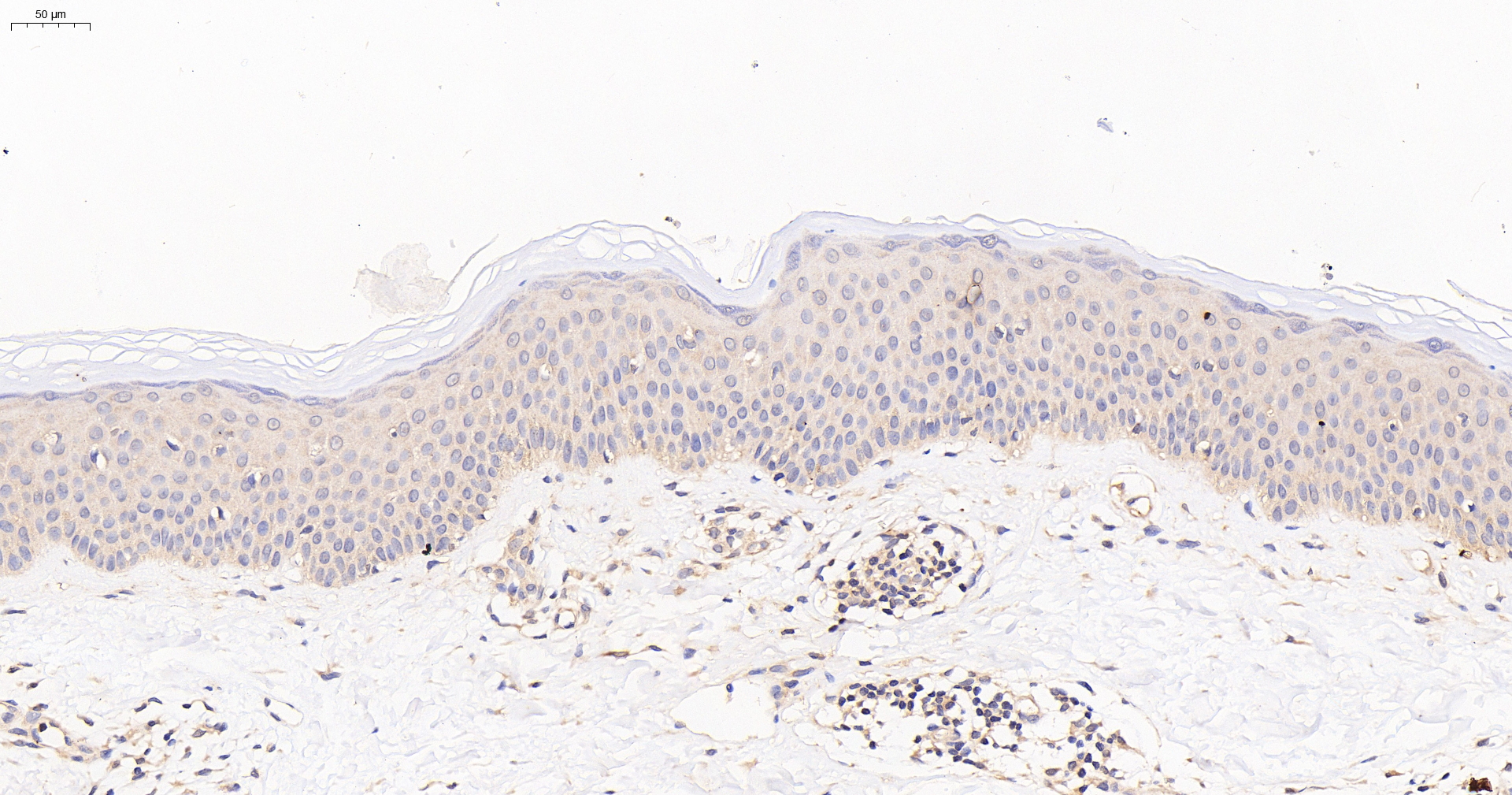

Supplement: Supplementary file 3 [file DataSheet1.zip › data/IHC/HC_1_20.0x1.jpg]

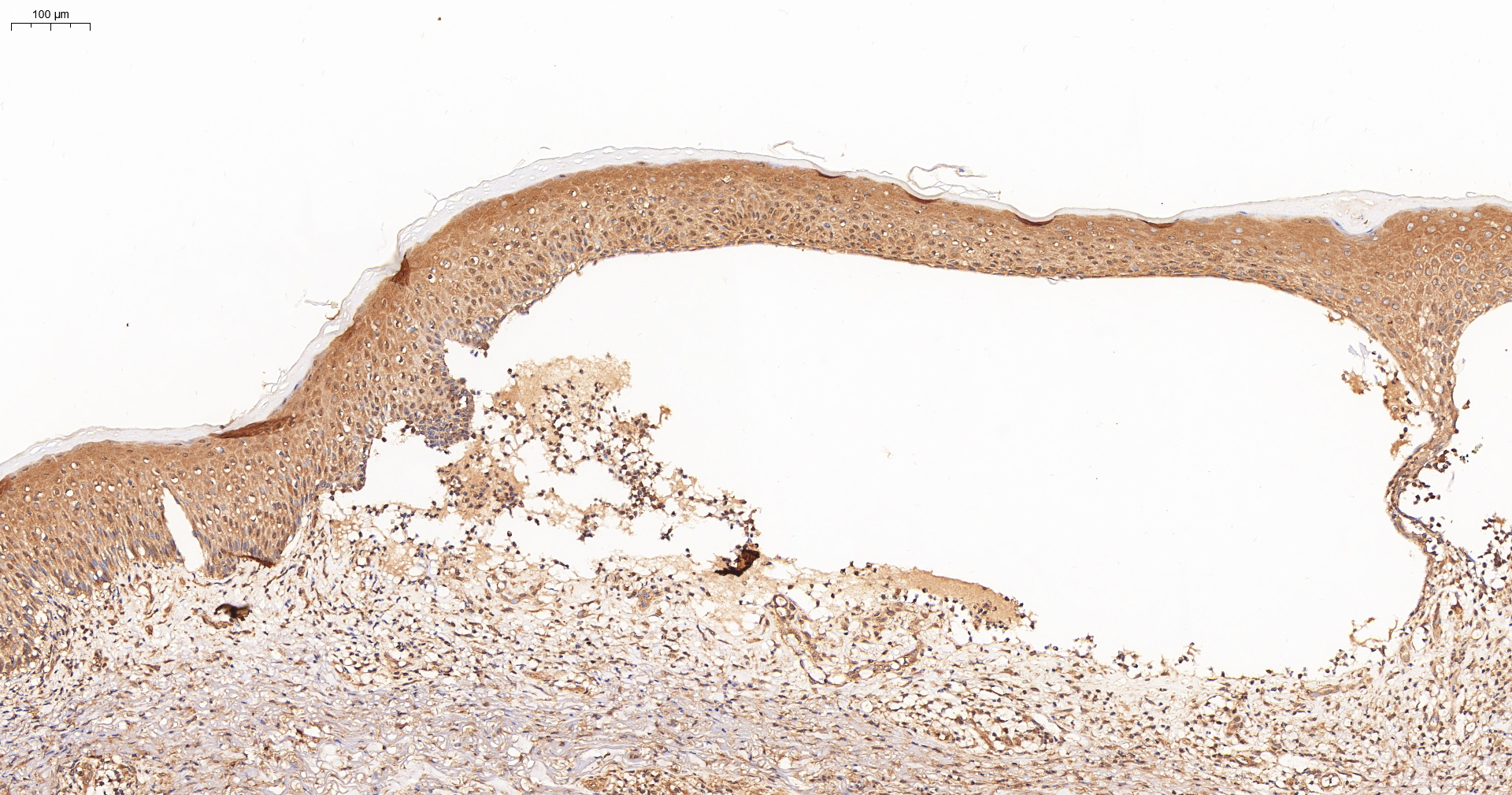

Supplement: Supplementary file 3 [file DataSheet1.zip › data/IHC/BP_1_10.0x.jpg]
