# Supplementary material for: IL-27 as a novel biomarker for pruritus in nodular prurigo and bullous pemphigoid
Source: Front Immunol. 2024 Dec 13;15:1499868. doi: 10.3389/fimmu.2024.1499868 (PMC11681427; doi:10.3389/fimmu.2024.1499868)
Supplement: Supplementary file 2 [file Table1.docx]

**Supplementary Table 1.** The feature of keg genes for SVM algorithms.

| **FeatureName** | **FeatureID** | **AvgRank** |
| --- | --- | --- |
| LINC01037 | 74 | 4 |
| PDCD6.DT | 121 | 9.2 |
| LOC107986784 | 97 | 11.4 |
| DCSTAMP | 35 | 14.4 |
| COL23A1 | 28 | 15 |
| AKAP12 | 5 | 16.8 |
| LOC105378571 | 92 | 18.6 |
| LOC105371792 | 86 | 19.4 |
| HDC | 54 | 22.2 |
| PTGER3 | 124 | 23.6 |
| LINC01637 | 76 | 24 |
| GCSAML | 50 | 24.4 |
| IL27 | 63 | 26.4 |
